# Effect of increased left ventricle mass on ischemia assessment in electrocardiographic signals: rabbit isolated heart study

**DOI:** 10.1186/s12872-017-0652-9

**Published:** 2017-08-04

**Authors:** Marina Ronzhina, Veronika Olejnickova, Tibor Stracina, Marie Novakova, Oto Janousek, Jakub Hejc, Jana Kolarova, Miroslava Hlavacova, Hana Paulova

**Affiliations:** 1 Department of Biomedical Engineering, Faculty of Electrical Engineering and Communication, Brno University of Technology, Technická 12, 616 00 Brno, Czech Republic; 2 Department of Physiology, Faculty of Medicine, Masaryk University, Kamenice 753/5, 625 00 Brno, Czech Republic; 3 Institute of Anatomy, First Faculty of Medicine, Charles University in Prague, U Nemocnice 3, 128 00 Prague, Czech Republic; 4 Institute of Physiology, Czech Academy of Sciences, Vídeňská 1083, 142 20 Praque, Czech Republic; 5 International Clinical Research Center, St. Anne’s University Hospital Brno, Pekářská 53, 656 91 Brno, Czech Republic; 6 Department of Biochemistry, Faculty of Medicine, Masaryk University, Kamenice 753/5, 625 00 Brno, Czech Republic

**Keywords:** Myocardial ischemia detection, Increased left ventricular mass, Electrogram, ROC analysis, Isolated heart, Rabbit

## Abstract

**Background:**

Detailed quantitative analysis of the effect of left ventricle (LV) hypertrophy on myocardial ischemia manifestation in ECG is still missing. The associations between both phenomena can be studied in animal models. In this study, rabbit isolated hearts with spontaneously increased LV mass were used to evaluate the effect of such LV alteration on ischemia detection criteria and performance.

**Methods:**

Electrophysiological effects of increased LV mass were evaluated on sixteen New Zealand rabbit isolated hearts under non-ischemic and ischemic conditions by analysis of various electrogram (EG) parameters. To reveal hearts with increased LV mass, LV weight/heart weight ratio was proposed. Standard paired and unpaired statistical tests and receiver operating characteristics analysis were used to compare data derived from different groups of animals, monitor EG parameters during global ischemia and evaluate their ability to discriminate between unchanged and increased LV as well as non-ischemic and ischemic state.

**Results:**

Successful evaluation of both increased LV mass and ischemia is lead-dependent. Particularly, maximal deviation of QRS and area under QRS associated with anterolateral heart wall respond significantly to even early phase (the 1^st^-3^rd^ min) of ischemia. Besides ischemia, these parameters reflect increased LV mass as well (with sensitivity reaching approx. 80%). However, the sensitivity of the parameters to both phenomena may lead to misinterpretations, when inappropriate criteria for ischemia detection are selected. Particularly, use of cut-off-based criteria defined from control group for ischemia detection in hearts with increased LV mass may result in dramatic reduction (approx. 15%) of detection specificity due to increased number of false positives. Nevertheless, criteria adjusted to particular experimental group allow achieving ischemia detection sensitivity of 89–100% and specificity of 94–100%, respectively.

**Conclusions:**

It was shown that response of the heart to myocardial ischemia can be successfully evaluated only when taking into account heart-related factors (such as LV mass) and other methodological aspects (such as recording electrodes position, selected EG parameters, cut-off criteria, etc.). Results of this study might be helpful for developing new clinical diagnostic strategies in order to improve myocardial ischemia detection in patients with LV hypertrophy.

## Background

Despite the intensive clinical and preclinical research, both morbidity and mortality associated with myocardial ischemia remain high. Diagnosis of myocardial ischemia might be complicated by co-incidence with other diseases, e.g. myocarditis, hypertension or left ventricular (LV) hypertrophy. The association between myocardial ischemia and LV hypertrophy has been intensely discussed during the last few decades. Particularly, the studies elucidated such important aspects as mechanisms of development and prevalence of myocardial ischemia in LV hypertrophy patients, specific character of analysis of ECG with ischemia-like patterns recorded in LV hypertrophy patients with and without evidence of myocardial ischemia, and others [1–3]. Nevertheless, detailed quantitative analysis of effect of LV mass changes on myocardial ischemia manifestation in ECG is still missing. Perhaps the only study, where the need of development of special criteria for ST elevation myocardial infarction in patients with LV hypertrophy was addressed, is study of Armstrong et al. [4]. Significantly different severity of ST elevation was obtained in LV hypertrophy patients (defined by standard voltage ECG criteria) with and without an angiographic culprit lesion. New diagnostic strategy based on standard criteria was proposed to improve specificity of ST elevation myocardial infarction detection (by decrease of false positive diagnoses) without loss of sensitivity.

In cardiovascular research, particular aspects can be successfully studied on animal models, frequently on isolated heart perfused according to Langendorff [5]. Although most of studies have been performed on rat heart [6–8], rabbit heart is more suitable. It represents optimal compromise between high level of similarity with human (in basic cardiac electrophysiology parameters, including ECG morphology [9], ionic channels distribution, process of repolarization, and calcium handling [10]) typical for big animal models on one side and easy breeding and low cost of small laboratory animals on the other side. Due to above benefits, rabbit is frequently used in ischemia studies [11, 12]. It also represents a suitable model for studying various aspects of LV hypertrophy, since high sensitivity to spontaneous LV hypertrophy (given by significant genetic factor and enhanced response to chronic stress) was previously reported in rabbit [13, 14]. Analogously, in our preliminary work, we reported spontaneous LV mass increase in rabbits [15]. For characterization of LV mass alteration, we introduced the term increased LV mass fraction, since the rabbit hearts did not meet generally accepted criteria of hypertrophy (evident developed structural changes of myocardium together with its electrical remodelling).

This paper presents the comprehensive study, where the effect of increased LV mass fraction on assessment of myocardial ischemia in electrocardiographic signals was addressed for the first time. Particularly, rabbit isolated heart model was used to: a) evaluate suitability of electrogram (EG) parameters for detection of increased LV mass fraction; b) quantify possible effects of increased LV mass fraction on the heart response to ischemia (in terms of onset, magnitude and reversibility of ischemia-induced changes in EG morphology); c) assess an impact of LV mass on efficiency of ischemia detection in EG. Besides the anatomical and electrical characteristics of the heart, the effect of mutual spatial orientation of the heart and electrode system on EG morphology was investigated, too. As a consequence, present work contributes to improving the quality and reliability of ischemia studies on animals and brings new information potentially useful for assessment of increased LV mass and myocardial ischemia.

## Methods

### Isolated heart preparation

All experiments were carried out with respect to recommendations of the European Community Guide for the Care and Use of Laboratory Animals and according to the experimental protocol approved by the Committee for Ensuring the Welfare of Experimental Animals, Faculty of Medicine, Masaryk University.

Sixteen adult New Zealand rabbits (both sexes, weight 2.2–3.45 kg) were included in the study. After premedication (diazepam i.m., 2 mg; heparin i.v., 1000 IU/kg), the rabbits were anaesthetized by mixture of xylazin (i.m., 2 mg/kg) and ketamin (i.m., 60 mg/kg). To prevent ischemia during heart preparation, trachea was cannulated and the animal was artificially ventilated (ventilator for small laboratory animals, World Precision Instruments, USA). Then the chest was opened, heart was rapidly excised and placed into cold (4 °C) Krebs-Henseleit (K-H) solution. The heart was fixed to a Langendorff apparatus and perfused with K-H solution (NaCl, 118 mM; NaHCO_3_, 24 mM; KCl, 4.2 mM; KH_2_PO_4_, 1.2 mM; MgCl_2_, 1.2 mM; CaCl_2_, 1.25 mM; glucose, 5.5 mM) aerated by pneumoxyd [16]. The temperature of perfusion solution and the perfusion pressure were maintained at 37 °C and 80 mmHg, respectively.

### Electrogram recording and experimental protocol

During the whole experiment, the heart was placed into the bath filled with the K-H solution and three EGs were recorded simultaneously by touch-less method using the orthogonal lead system [17]. It included Ag-AgCl disc electrodes placed in the inner wall of the bath. The signals were amplified by a set of three biological amplifiers DAM50 (World Precision Instruments, USA) and further simultaneously digitized by 16-bit AD converters at a sampling rate of 2000 Hz using a data acquisition multifunction card PCI-6250 (National Instruments, USA).

**Fig. 1:**
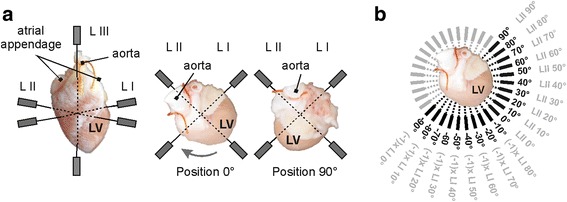
Electrogram recording: **a** orthogonal system of electrodes on front (*left*) and top (*middle* and *right*) view; **b** scheme of EG reconstruction in the range from −90° to +90° (*black font*) using data recorded with two horizontal bipolar leads during the heart rotation from 0° to 90° (*grey font*); opposite electrodes of the bipolar leads are depicted with *grey boxes* on top view. LV – left ventricle; L – lead; *grey arrow* – direction of rotation. Lead III is not shown on top views

The experimental protocol consisted of stabilization (25 min long), the rotation of the heart (5 min), global ischemia (induced by cessation of perfusion), and reperfusion (each 10 min long). In stabilization period, the hearts were rotated around their longitudinal axis from 0° to 90° in 10° steps, where 0° was considered as initial heart position in recording system (anterior wall facing forward, see Fig. 1a). EG recorded in each step of rotation included 10–20 QRS complexes. EGs in positions −90° to 0° were subsequently reconstructed using data recorded by lead I and lead II during rotation of the heart within the range 0° to 90° (see Fig. 1b). During ischemia and reperfusion, initial position of horizontal leads was chosen for EGs recording.

### Direct assessment of increased LV mass

Before anaesthesia, body weight (BW) of each animal was assessed. Immediately after the isolated heart experiment, the whole heart was weighted (heart weight, HW). Both atria and right ventricle were then separated and LV with septal wall was weighted (LV weight, LVW). Free lateral wall of LV was cut and the wall thickness (LVT) was measured.

To assess spontaneously changed anatomical characteristics of the heart, following indexes were calculated: the heart weight to body weight (HW/BW) ratio, the LV weight to body weight (LVW/BW) ratio and LV weight to heart weight (LVW/HW) ratio. According to the results of retrospective analysis, LVW/HW ratio – representing the LV fraction in the whole heart mass – was the only index suitable for dividing the animals into two groups. The discrimination threshold value of LVW/HW ratio (0.57) was found by analysis of receiver operating characteristics (ROC) curve [18]. Thus, animals with LVW/HW ratio below or equal threshold were assigned to group L and animals with the ratio above threshold to group H (LVW/HW 0.53 ± 0.03 and 0.61 ± 0.03, respectively; *p* < 0.001, Mann-Whitney U test; *n* = 8 for both groups).

### Electrogram parameters calculation

**Fig. 2:**
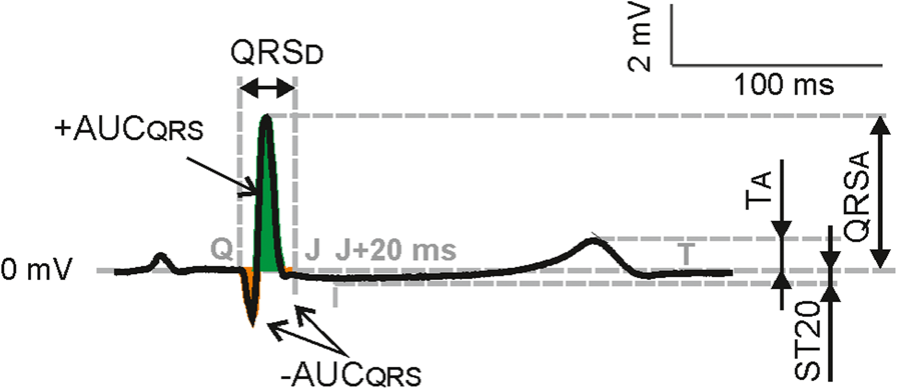
Electrogram parameters calculation: QRS_D_, QRS_A_ – duration and absolute maximal deviation of QRS, respectively; +AUC_QRS_, −AUC_QRS_ – area under positive and negative part of QRS, respectively; T_A_ – maximal deviation of T wave; ST_20_ – level of ST segment 20 ms after QRS offset. Positive and negative parts of QRS also contribute to calculation of area under whole QRS (AUC_QRS_, not shown) positively and negatively, respectively

Before EG parameters calculation, the EG segments with artefacts were excluded from the analyses. The low-frequency baseline wander was suppressed using Lynn’s filter with cut-off frequency of 0.5 Hz. After filtering, QRS complexes were automatically detected using wavelet based detector. For representation of particular lead positions (see Fig. 1b), averaged QRS-T were calculated from highly correlated (Spearman’s ρ more than 0.99) subsequent segments. For further processing, the beginnings and the ends of QRS complexes and the end of T wave were detected. Common and newly proposed QRS- and ST-T-related parameters (see Fig. 2) were evaluated in EGs recorded during rotation and during short-term global ischemia and reperfusion in both groups.

Following QRS-related parameters were analysed in each rotation position in stabilization period: QRS duration (QRS_D_), absolute value of maximal QRS deviation (QRS_A_), area under whole QRS (AUC_QRS_, AUC - area under curve; positive and negative parts of QRS contribute to AUC calculation positively and negatively, respectively), and area under positive (+AUC_QRS_) and negative (−AUC_QRS_) part of QRS. These parameters were also calculated from EG recorded within the whole ischemia and reperfusion by initially placed lead I and lead II (see above).

Besides abovementioned parameters, level of ST segment at J + 20 ms point (ST20) and maximal deviation of T wave (T_A_) were calculated. The former was chosen empirically as an alternative to ST60 used in human ECG analysis considering the differences in characteristics (mainly QT and ST-T duration) of human ECG and EG of rabbit isolated heart.

### Statistical analysis of data

It was found, that data are not normally distributed (Shapiro-Wilk test). The non-parametric Mann-Whitney U-test was then used to test the differences between L and H groups in following data sets: a) EG parameters in different heart positions calculated in stabilization; b) EG parameters calculated from data recorded during ischemia and reperfusion. Additionally, Wilcoxon signed rank test was applied in L and H group separately in order to reveal possible significant changes of the parameters appeared at the end of each minute during ischemia and reperfusion (as compared to stabilization values). Correlation between anatomical characteristics was investigated using Spearman’s correlation coefficient *ρ*. The ability of different parameters to detect the increased LV mass fraction or/and ischemia-induced EG changes was investigated by ROC analysis. The sensitivity (Se), specificity (Sp), area under ROC curve (AUCROC), and optimal cut-off point were used to quantify the detection performance. Particularly, AUCROC of 0.5–0.6 and 0.9–1 represents poor and excellent discrimination performance of the parameter, respectively [18]. For all abovementioned tests, *p* < 0.05 was considered as significant.

## Results

### Electrophysiological effects of increased LV mass fraction under non-ischemic condition

**Fig. 3:**
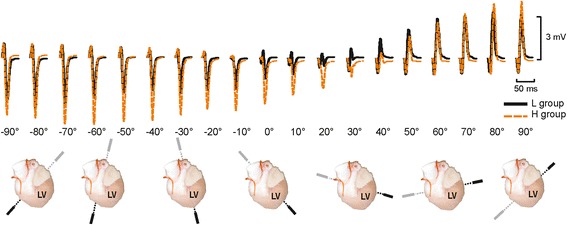
Averaged QRS complexes recorded in hearts with low (L) and high (H) LV mass fraction in the range from −90° to +90° (*top*). Top views of the heart illustrate the position of bipolar lead during electrogram recording (*bottom*). LV – left ventricle

**Fig. 4:**
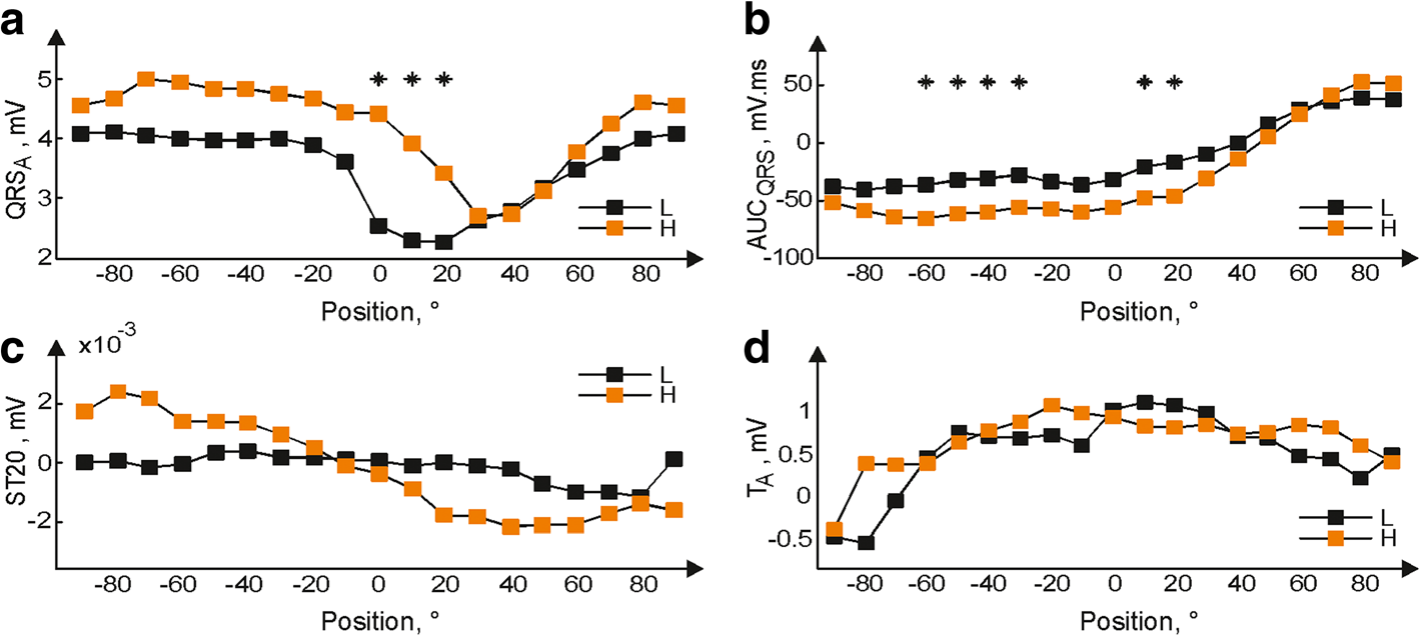
Median values of QRS_A_ (**a**), AUC_QRS_ (**b**), ST20 (**c**), and T_A_ (**d**) calculated from electrograms recorded in hearts with low (L) and high (H) LV mass fraction in the range from −90° to 90° (* *p* < 0.05)

Courses of averaged QRS complexes calculated for L and H group in various heart positions are shown in Fig. 3. In some positions, morphology of QRS evidently varies among the groups. It corresponds with the results of statistical analysis of QRS-related parameters, where the significant differences between the groups were found in QRS_A_ (in the range of <+0°, +20°>) and in AUC_QRS_ (in the range of <−60°, −30° > and <+10°, +20°>) (see Fig. 4a-b). No significant differences were found in ST-T parameters, including ST20 and T wave polarity (Fig. 4c-d).

### Assessment of increased LV mass fraction by EG morphology analysis

**Fig. 5:**
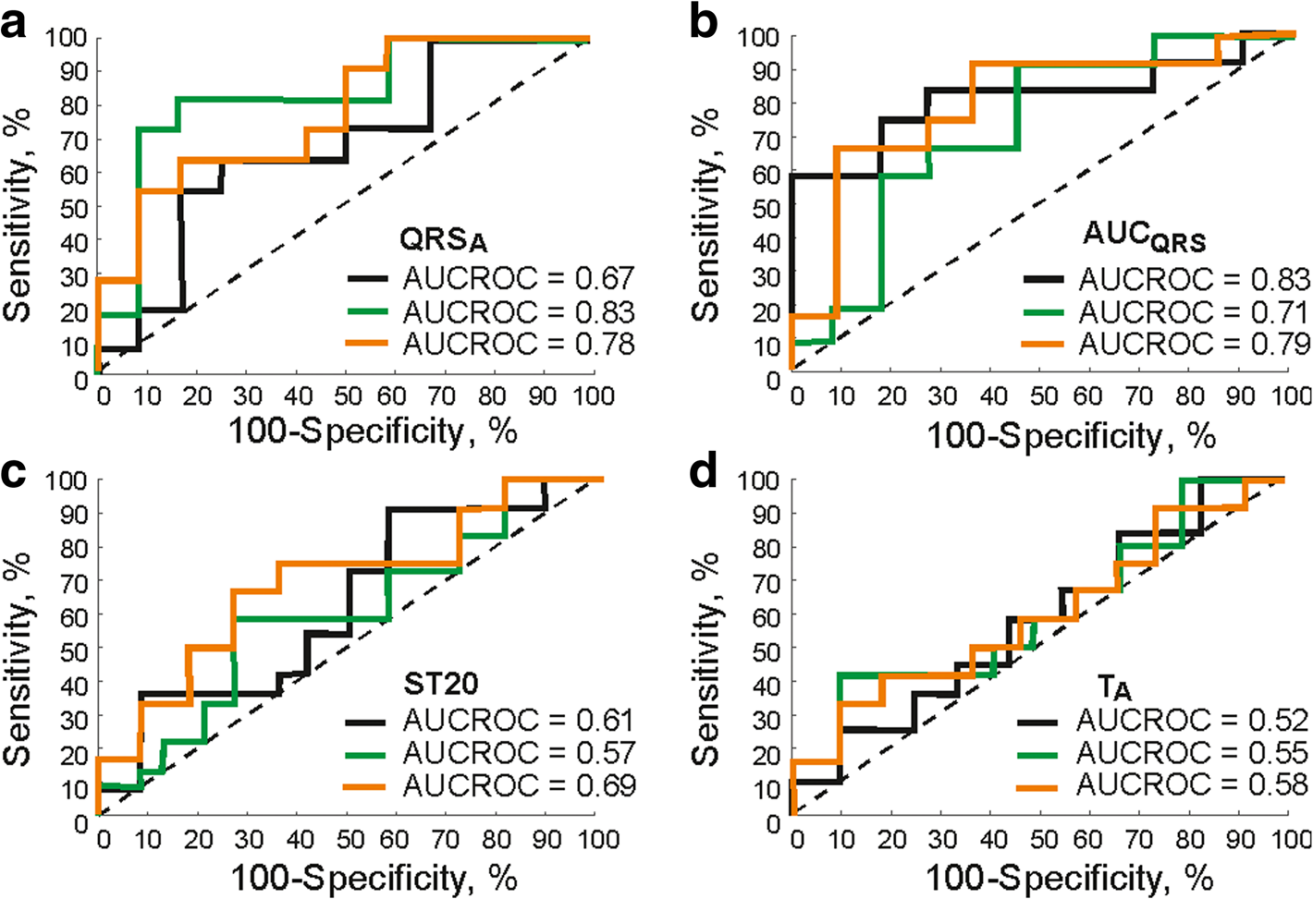
Receiver operating characteristics curves calculated for QRS_A_ (**a**), AUC_QRS_ (**b**), ST20 (**c**), and T_A_ (**d**) in the positions −30°, 0°, and 20° (*black*, *green* and *orange*, respectively) for the detection of increased LV mass fraction. AUCROC – area under receiver operating characteristics curve

Above findings are in accordance with the results of ROC analysis used for evaluation of the ability of different parameters to detect increased LV mass fraction. As seen in Fig. 5a-b, the best diagnostic performance of QRS_A_ and AUC_QRS_ indicated by the highest AUCROC was achieved in the position 0° and −30°, respectively. Corresponding performance indices for QRS_A_ (AUC_QRS_) were: cut-off point 3.3 mV (−45.3 mV·ms), Se 82% (75%) and Sp 83% (82%). Indices calculated for other parameters were significantly lower within the whole recording range (Fig. 5c-d).

### Electrophysiological effects of ischemia on the hearts with low and high LV mass fraction

Firstly, magnitude and time onsets of ischemia manifestations were evaluated in each group separately. Parameters measured at the end of each minute of ischemia were compared with those from the end of stabilization by paired test. Onsets of significant ischemia-induced changes of different parameters are summarized in Table 1. In all parameters, the increase of the values (with maximal elevation at the end of ischemia) was revealed. Generally, onsets of significant changes in both groups were the same; the earliest prominent changes (already in the 1st minute of ischemia) appeared in QRS parameters, mostly derived from lead II. In case of QRS_A_ and -AUC_QRS_, the changes were indicated only in lead II. In contrast to QRS parameters, those calculated from ST-T increased significantly only in the second half (the 5^th^ minute or later) of ischemia. In lead I data, ischemia manifestations were generally indicated with 1–2-min delay as compared to lead II. Values of almost all parameters returned to the control level immediately at the beginning of reperfusion. The exceptions were QRS_D_ and ST20 from H group, where ischemia-induced changes disappeared after 3–4 min of perfusion recovery.

**Table 1:** Onset of significant (*p* < 0.05) ischemia-induced changes in EG parameters. L, H – hearts with low and high LV mass fraction, respectively; ‘-’ – no significant changes

Parameter	Onset, min
QRS_A_, AUC_QRS_, and -AUC_QRS_ (L and H, lead II) +AUC_QRS_ (L and H, lead I)	1st
QRS_D_ (L and H) AUC_QRS_ (L and H, lead I) +AUC_QRS_ (L and H, lead II)	3rd
ST20 and T_A_ (L and H, lead I)	5th
ST20 and T_A_ (H, lead II)	6th
ST20 and T_A_ (L, lead II) QRS_A_ and -AUC_QRS_ (L and H, lead I)	-

**Fig. 6:**
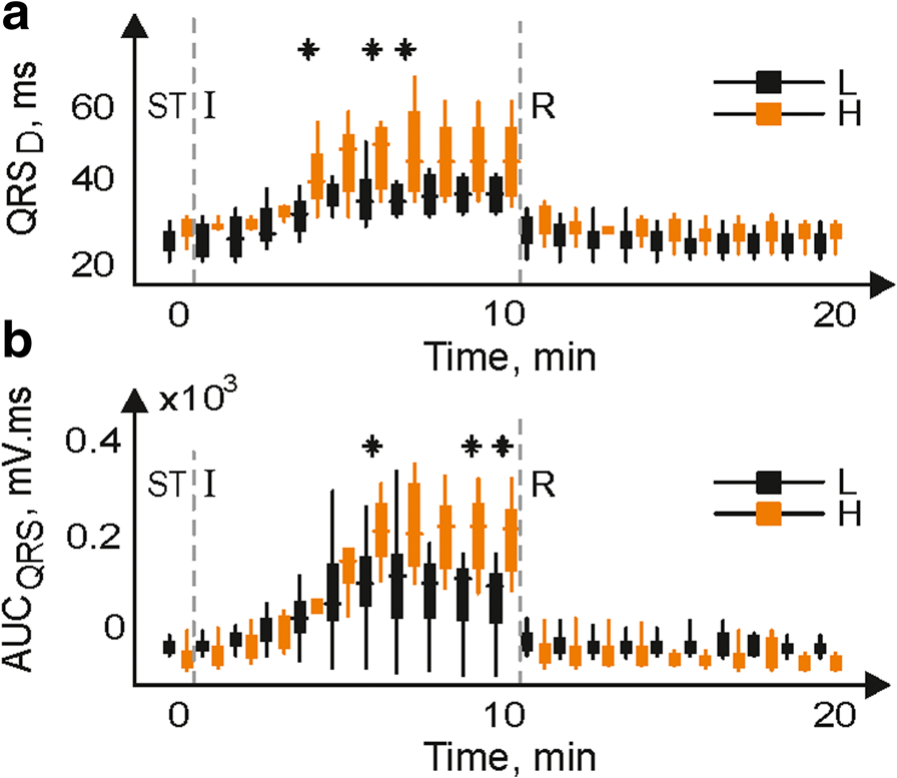
Distribution of QRS_D_ (**a**) and AUC_QRS_ from lead I (**b**) in hearts with low (L) and high (H) LV mass fraction during the end of stabilization (ST), ischemia (I) and reperfusion (R) (* *p* < 0.05). Inside band, *top* and *bottom of boxes* indicate median, 25th and 75th percentiles, respectively

Secondly, the responses of the hearts with unchanged and increased LV mass fraction to myocardial ischemia and reperfusion were compared via comparison (using unpaired test) of parameters measured in both groups during corresponding experimental periods. Significant differences were found in case of QRS_D_ (4^th^–7^th^ min of ischemia) and AUC_QRS_ and +AUC_QRS_ (5^th^–10^th^ min of ischemia). In Fig. 6, the distribution of QRS_D_ and AUC_QRS_ in each minute of corresponding experimental period is shown using box plots. It is evident that above differences are due to accentuation of parameters values in H group. No differences were found in values of ST-T parameters during ischemia as well as in all parameters calculated from reperfusion.

### Effect of LV mass fraction on myocardial ischemia detection

**Fig. 7:**
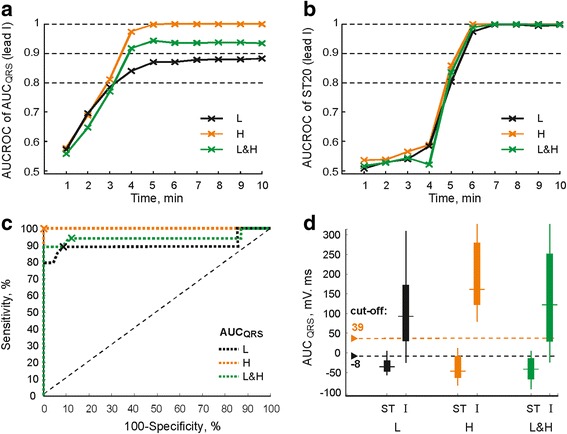
Area under receiver operating characteristics curve (AUCROC) for AUC_QRS_ (**a**) and ST20 (**b**) during ischemia; ROC curves for AUC_QRS_ at the end of ischemia (**c**); distribution of AUC_QRS_ in stabilization (ST) and at the end of ischemia (I) and corresponding discriminating cut-off values (**d**). L, H, L&H – hearts with low and high LV mass fraction and united group, respectively

Above results of paired statistical test are in agreement with those of ROC analysis, which was used to evaluate the overall ability of the parameters to discriminate between non-ischemic and ischemic state. For example, in case of parameters with the earliest response to ischemia (such as QRS_A_ derived from lead II or AUC_QRS_ derived from lead I or lead II in both groups, etc.), AUCROC reaches 0.8 (indicating good discrimination ability) in approx. The 3^rd^-5^th^ minute of ischemia (see Fig. 7a). For parameters with delayed response to perfusion cessation (e.g. ST20, except for that calculated from lead II in L group), such a good discrimination between non-ischemic and ischemic data can be obtained in approx. The 5th–8th minute of ischemia (see Fig. 7b). In most parameters calculated in both groups, AUCROC increases up to 0.98–1 (perfect discrimination) at the end of ischemia with corresponding Se and Sp of approx. 75% - 90%, even 100% in some cases (similar to abovementioned AUC_QRS_ and ST20, see Fig. 7a-c).

The effect of LV mass on criteria and accuracy of myocardial ischemia detection can be illustrated by Se, Sp and cut-off calculated for different experimental groups from corresponding ROC curves. For example, the differences in AUC_QRS_ between groups L and H appearing in approx. The 4th minute of ischemia (see Fig. 6b) are reflected in corresponding courses of AUCROC (L and H in Fig. 7a). Results of ROC curve analysis for lead I AUC_QRS_ calculated at the end of ischemia are summarized in Table 2. As can be seen, the cut-offs used to distinguish between non-ischemic and ischemic data (also depicted with arrows in Fig. 7d) and corresponding Se and Sp (operating points with optimal cut-off value on ROC curves are also depicted with crosses in Fig. 7c) obtained in particular groups are quite different. In particular, the cut-off value in L group is negative, whereas that of H group is positive. Se and Sp in H group reach maximal possible value (100%). Following observations were made from detailed investigation of ROC analysis results.

**Table 2:** Performance indices of myocardial ischemia detection (the 10th minute of ischemia) using lead I AUC_QRS_ calculated for hearts with low (L) and high (H) LV mass fraction and united group (L&H)

Analysed group	Se, %	Sp, %	Cut-off, mV·ms	Group used for cut-off calculation
L	89	94	−8*	L
H	100	100	39*	H
L&H	94	90	−8*	L&H
L	79	98	39	H
H	100	85	−8	L
L&H	89	99	39	H

Se – sensitivity; Sp – specificity; * – optimal (‘adjusted’) discriminating cut-off derived from ROC curve for corresponding group

Use of L group cut-off (−8 mV·ms) for ischemia detection in the hearts with high LV mass fraction results in prominently decreased Sp (by approx. 15%) as compared to detection based on H group cut-off (39 mV·ms) due to increased number of false positives (see H in Fig. 7d). On the contrary, the cut-off previously calculated from H group data should not be used for ischemia detection in L group because of low Se (only 79%) obtained in this case due to dramatically increased number of false negatives (see L in Fig. 7d).

If data from the hearts with low and high LV mass fraction are analysed together (united group, L&H in Table 2), slightly higher Se (by 5%) and lower Sp (by 4%) are obtained comparing with the indexes calculated for L group using the same cut-off (−8 mV·ms). Decrease of both Se (by 6%) and Sp (by 10%) is indicated in case of united group analysis as compared to evaluation of H group separately using corresponding cut-off (39 mV·ms).

Use of H group cut-off (instead of that calculated from L and H data together) for ischemia detection in united data results in decreasing of Se (by 5%) and increasing of Sp (by 9%) due to increased number of false negatives and decreased number of false positives, respectively (see L&H in Fig. 7d).

Similar tendencies were obtained for +AUC_QRS_ and QRS_D_.

## Discussion

### LV mass alteration and its direct assessment

In contrast to the human, where LV mass assessment is generally based on evaluation of LV volume parameters (measured by echocardiographic or magnetic resonance imaging and subsequently normalized to body weight, body surface area, body mass index, or height [19–21]), in animal studies, LV mass and other anatomical parameters (e.g. LVW/BW ratio [22–24]) can be measured directly. Slight LV mass changes cannot be detected using LVW/BW ratio. Therefore, more sensitive index − the LVW/HW ratio representing the mass fraction of LV in the whole heart mass − was used in this study for evaluation. According to statistical analysis, the LVW/HW ratio is the only index affected with slight increase of LV mass. Normalization of LVW by HW instead of BW seems to be reasonable because of high correlation (Spearman’s ρ) between HW and BW (0.72, *p* < 0.05), LVW and BW (0.86, *p* < 0.05) and LVW and HW (0.92, *p* < 0.01).

Increased LV mass fraction in group H was accompanied by insignificant but clearly visible increase of collagen content (evaluated by Masson trichrome staining) together with decreased tolerance of such myocardium to ischemic insult, as was previously reported [15]. Considering absence of infectious disease (all rabbits used in our study were purchased from certified supplier – Velaz Ltd., Czech Republic – with all appropriate health clarity declarations) and stress insults (animals were handled according to the European Community Guide for the Care and Use of Laboratory Animals) and according to previously reported predisposition of the rabbits to spontaneous LV hypertrophy [13, 14], we hypothesize that abovementioned observations are early signs of spontaneously developing LV alteration.

### Reliability of EG recording in various heart positions

One of the factors influencing the quality and characteristics of ECG is the mutual orientation of electrode system and the heart which can be affected with the subject’s body position or electrode placement during recording procedure. Clinically significant ST segment deviation and changes of QRS complexes (polarity and relative size of Q, R and S) in standard ECG recorded at right and left-side lying position (comparing with supine) in both healthy and subjects with cardiac disease were reported [25]. The dramatic worsening of detection of ischemia and LV hypertrophy caused by variation in the positioning of chest electrodes was reported as well [26].

In the present study, longitudinal rotation of the heart placed in the bath filled with K-H solution was performed in stabilization in order to obtain EGs from different sites of LV. In both animal groups, no significant “pseudo-ischemic” alterations (such as deviation of ST segment, change of polarity or increase of T wave amplitude, and widening of QRS complex) were found in EGs, including those from initial position of horizontal leads, which is suitable for monitoring the changes in EG morphology caused by global ischemia [27]. Small alterations in ST20 in boundary LV area of H group data (Fig. 4c) were negligible in comparison with ischemia-induced ST deviation observed in both groups (at least 2 mV at the middle of ischemia). Thus, detection of increased LV mass fraction or ischemia in this data should not be affected with the heart orientation.

### Ability of EG parameters to detect increased LV mass fraction

It is known that anatomical changes of the heart such as LV hypertrophy produce the changes in ECG morphology including increased amplitude and QRS complex prolongation, QRS patterns associated with the defects of intraventricular conduction and the left axis deviation [1, 28, 29]. Despite relatively low sensitivity of ECG-based LV hypertrophy detection (in the range of 40–60%), electrocardiography is still frequently used for LV hypertrophy screening due to its low cost, easy performance and wide availability [30, 31]. Many electrocardiographic indexes have been proposed for diagnosis of LV hypertrophy in human. Most of them are based on the so-called QRS voltage criteria mainly utilizing S and R peak amplitudes in the decision procedure [1, 31]. Thus, diagnostic performance of these indexes particularly depends on the precision of QRS complexes delineation. However, in clinical as well as experimental data, the detailed delineation of QRS is challenging task, especially in case of EG recorded under variable conditions [32].

This study reports that even slight change of LV size (manifested in neither LVW/BW ratio nor significantly changed structure of myocardium such as in case of developed LV hypertrophy) can be accurately detected using easily calculated EG parameters (without the need of complete delineation of all parts of QRS complex). As in human [1], one of such parameters is QRS_A_. Nevertheless, AUC_QRS_ seems to be the most sensitive to the changes in electrical activity caused by LV mass fraction increase (see Fig. 5). This is probably due to the method of parameter calculation, where all peaks within the whole QRS complex are taken into account including their polarity. As a result, minor changes in QRS morphology cause significant change of AUC_QRS_ value (compare graphs in Fig. 3 and Fig. 4b). It allows to detect increased LV mass fraction with relatively high Se and Sp (both approx. 82%, see Fig. 5b). However, it should be stressed that higher success of presented approach as compared to clinical diagnostics based on routine ECG is most likely due to experimental, detailed type of analysed data (EG from isolated heart with no effects of neurohumoral regulation and muscle activity, well conductive K-H solution in a space between the heart surface and electrodes instead of conduction inhomogeneity such as in torso, carefully selected electrodes positions, and fixed distance between the heart surface and electrodes to reduce the inter-subject variability). Thus, method for LV hypertrophy assessment in experimental model cannot be easily applied to human data. On the other side, there are certain similarities in observations from both types of data. For example, above finding regarding diagnostic capacity of AUC_QRS_ is in a good agreement with the results of study on ECG recorded in healthy subjects and patients with LV hypertrophy, where significant improvement of diagnostic accuracy was achieved by using voltage-duration product and true time-voltage QRS area instead of common QRS voltages and duration [33]. It should be also noted that areas with high accuracy of increased LV mass fraction detection in rabbit isolated heart electrogram (<−60°,-30° > and <0°, 20°>) correspond roughly with areas usually used for LV hypertrophy detection in human ECG (precordial leads V1, V2, V5, and V6 [1]).

### Electrocardiographic detection of myocardial ischemia with respect to LV mass fraction

According to ESC/ACCF/AHA/WHF, the earliest ischemia-induced changes in human are reflected in ECG on T wave and ST segment and the changes in QRS complex are generally associated with severe myocardial ischemia (eventually myocardial infarction) [34]. These changes are linked to the region of myocardial ischemia and, thus, can be used to its localizing. In the present study, the model of global myocardial ischemia was used. Although the regional ischemia is in the centre of interest in clinical practice, global ischemia is preferred in studies on rabbit hearts due to its simplicity and high reproducibility regardless of inter-subject differences in anatomy of coronary system and presence of collateral flow [11, 35]. In contrast to the regional ischemia in human, the earliest ischemia-induced changes in the rabbit model are associated with electrical activity during ventricular depolarisation. In both experimental groups, it is mainly reflected in the values of QRS-related parameters extracted from EG recorded with lead oriented approximately through the anterolateral wall of LV (lead II at initial position in Fig. 1). Some data from this area, however, seem to be sensitive to LV mass increase, too (Fig. 4a-b and Fig. 5a-b). If electrophysiological effects of LV mass increase on investigated phenomena (such as developed myocardial ischemia, etc.) are not desired, only parameters resistant to such effects (such as +AUC_QRS_ and -AUC_QRS_) should be included in the study. Other possible approach is use of data recorded from boundary LV areas (near the initial position of lead I in Fig. 1), where no significant effect of LV mass on the parameters were found (see Fig. 4 for various parameters in stabilization and Fig. 7b for ST20 in ischemia). However, ischemia-induced changes in such case can be revealed with some time delay compared to previous one (see Table 1). Thus, appropriate parameters and/or recording area should be carefully chosen depending on the study goal.

It is worth mentioning, that even recording with leads ‘insensitive’ to LV mass fraction increase in stabilization period does not ensure that EG alterations indicated in ischemia are associated merely with this pathological condition. It is because the influence of LV anatomical change on the heart electrical activity may become apparent during ischemia. This may be explained by certain electrical dissynchrony based on subtle metabolic changes in hearts from H group. Escalation of electric inhomogeneity could be attributed to aggravation of impaired oxygen supply in the heart with slightly increased LV mass by acute ischemic insult. In case of myocardial hypertrophy, impaired supply of oxygen results from increased cardiomyocyte dimensions, perivascular infiltration of coronary arteries, altered secretion of endothelial derived factors, etc. [36, 37]. Inadequate supply of oxygen in such hearts was revealed by ischemic insult only and led to accentuating manifestation of ischemia. Such mechanism might be responsible for significant difference in QRS_D_ and AUC_QRS_ parameters between L and H groups revealed in the middle of ischemia (Fig. 6), though only insignificant structural changes of the myocardium in group H were reported [15].

As mentioned above, this phenomenon may have an impact on ischemia assessment, where the detection accuracy depends directly on discriminating cut-off. Use of unsuitable cut-off (e.g. if its value is calculated from the group different than that being analysed) obviously results in increase of false positive or false negative detections and, consequently, in decrease of Sp or Se (or both) (see Table 2 and Fig. 7d). Reduced quality of ischemia detection is particularly expected, if no attention is paid to LV mass and data derived from L and H groups are analysed together. It results in underestimation of the detection performance as compared to that obtained in L and H group, when the cut-offs ‘adjusted’ to corresponding groups are used. In some cases, adjusted cut-offs provide perfect results with Se and Sp of 100% (H in Table 2). Thus, the cut-off value should be carefully set with regard to the type of analysed data. It is generally in agreement with the studies, where increase of false positive detections due to neglecting of various patient-related factors (e.g. gender, age, LV hypertrophy, etc.) affecting ECG morphology at rest was revealed and adaptation (arising) of ST-segment cut-off was suggested to improve ischemia detection accuracy [34, 38]. Analysis of anatomical peculiarities of the heart may help to reduce number of incorrect detections and avoid confusions in results interpretation.

## Conclusions

In this study, it was shown that coincidence of LV mass alteration and myocardial ischemia leads to accentuation of some patterns in ECG, as compared to manifestations of ischemia with any other concomitant pathology. Since this phenomenon is reflected in values of ECG parameters, cut-off based criteria for ischemia detection must be chosen with a caution taking into account anatomical characteristics of LV. Neglecting this aspect may lead to dramatic decrease of ischemia detection accuracy.

Despite experimental character of this study, some methodological aspects and issues addressed (e.g. regarding selection of appropriate ECG parameters and recording electrodes position, method for quantitative evaluation of the effect of altered LV on ischemia detection criteria and detection performance, etc.) can be considered relevant for clinical practice due to similarities in characteristics of rabbit and human heart. Particularly, results of this study might be helpful for improvement of myocardial ischemia detection in patients with LV hypertrophy.

## Abbreviations

AUCROC: Area under ROC curve
BW: Body weight
EG: Electrogram
HW: Heart weight
LV: Left ventricle
LVT: LV thickness
LVW: LV weight
ROC: Receiver operating characteristics
Se: Sensitivity
Sp: Specificity
